# The Foreign Journals: Medical Statistics

**Published:** 1839-07

**Authors:** 


### MEDICAL STATISTICS.

*Report of the Results of Revaccination in the Prussian Army in the year* 1838, *drawn up from Official Documents.* By Dr. LOHMEYER.

TOTAL number of individuals inoculated (revaccinated) 42,041, of which number there were cicatrices from former vaccination

| | |
|---|---|
| distinct, in . . . . | 33,819 |
| indistinct, in . . . | 5,645 |
| none, in . . . . . | 2,577. |
| The resulting vaccination was regular in . . | 19,117 |
| irregular in . | 8,672 |
| did not take in . | 14,252. |
| In the cases of failure the vaccination was repeated, | |
| with effect in . . . | 2,306 |
| without effect in . . | 10,424. |

Of the individuals in whom the disease was perfectly regular, there were from

| | | | |
|---|---|---|---|
| 1 | to 5 | pustules in | 8,787 |
| 6 | .. 10 | ...... | 5,581 |
| 11 | .. 20 | ...... | 4,056 |
| 21 | .. 30 | ...... | 693. |

Of individuals revaccinated with effect in the present and former years, there were affected, during 1838,

| | |
|---|---|
| with varicella . . . | 19 |
| .. varioloid . . . | 10 |
| .. true smallpox . . | 2 |

It appears, from the preceding statement, that the results of revaccination in the army, during the year 1838, closely resemble those observed in 1837, (see *British and Foreign Med. Rev.* vol. VII. p. 186;) in both years about 45 in the 100 exhibiting true vesicles running a regular course. The resemblance would have been still greater, but for the circumstance that many men were included who, according to their own account, had been previously revaccinated at their own homes, but who exhibited no marks on their arms; these were almost always among the failures. According to the reports of the medical officers, there were also many cases in which the development and course of the disease were disturbed, chiefly through carelessness on the part of the individuals in allowing the pustules to be broken or otherwise injured.

The opinion formerly pretty prevalent that, failing lymph immediately from the cow, the only proper matter for vaccination is that taken from children with good pocks, has now few supporters among the military surgeons, as they have ascertained by manifold experience that the lymph from well-formed pocks of adults, whether in the first or second vaccination, produces as fine and regularly-proceeding vesicles as that from children. Accordingly, it is the practice of most of the medical officers, only in the commencement of the revaccination to use lymph from children. Many even, like the Wurtemburg vaccinators, give the preference to the latter for communicating the disease to adults.

Inoculation with dry, preserved lymph, was in a great degree ineffectual. In a child vaccinated with lymph of this kind, there appearing no result, it was again vaccinated, eight days later, with fresh lymph from the arm, after which not only all the latter punctures, but also two of the former took and exhibited good and regularly-proceeding pocks. In the case of a man of the 6th artillery brigade, the pocks did not appear until six weeks after the inoculation (two on the right and three on the left arm), and their development was accompanied by so much inflammation in the vicinity, and gave rise to so much fever, that it became necessary to remove the patient into the hospital.

In several individuals natural smallpox appeared soon after the vaccination. In two cases this happened before the third day, and in these the vaccination had no result. In two other cases, on the contrary, the modified smallpox (varioloid) appeared when the vaccine vesicles were in perfection, and then they sustained no alteration in their progress.

The whole number of smallpox cases in the army, in 1838, were 111, 56 being characterized as varicella, 43 as varioloid, and 12 as true variola; in this number are included the 31 cases formerly mentioned as occurring after successful revaccination. Seven cases of the smallpox were fatal, but no fatal cases occurred among the 31 just mentioned; in all of whom, on the contrary, the disease was mild, and indeed quite insignificant. The greater number of cases of smallpox (as was also the case in former years) took place in recruits shortly after joining the army, and before revaccination could be employed. Some of the older soldiers, however, were also attacked with the disease in some of its forms; for instance, some of the subordinate officers who had been previously revaccinated without effect, or who had entered the army previously to the introduction of the practice of revaccination. Most of the cases of natural smallpox occurred in the 7th division, viz. 37, and for the most part in the garrison of Minden. In the 4th division not a man failed with smallpox, although the troops mixed more or less with the inhabitants of their stations or of the vicinity. This favorable result is principally to be attributed to this circumstance—that in the district of the 4th division the recruits joining the army in the autumn of 1837 were, for the most part, subjected to revaccination immediately on their arrival; whereas, in other cases, it has been customary to put off the revaccination of such recruits, at least in a considerable proportion, until the spring. *Medicinische Zeitung. May* 8, 1839.

---

### II. THE AMERICAN AND COLONIAL JOURNALS.

### PATHOLOGY, PRACTICAL MEDICINE, AND THERAPEUTICS.

